# Effects of diaphragm stretching on posterior chain muscle kinematics and rib cage and abdominal excursion: a randomized controlled trial

**DOI:** 10.1590/bjpt-rbf.2014.0169

**Published:** 2016-06-16

**Authors:** Francisco J. González-Álvarez, Marie C. Valenza, Irene Torres-Sánchez, Irene Cabrera-Martos, Janet Rodríguez-Torres, Yolanda Castellote-Caballero

**Affiliations:** 1 Department of Physical Therapy, University of Granada, Granada, Spain

**Keywords:** muscle stretching exercises, diaphragm, movement, physical therapy

## Abstract

**Background:**

Few studies have explored the effects of stretching techniques on diaphragm and spine kinematics.

**Objective:**

To determine whether the application of diaphragm stretching resulted in changes in posterior chain muscle kinematics and ribcage and abdominal excursion in healthy subjects.

**Method:**

Eighty healthy adults were included in this randomized clinical trial. Participants were randomized into two groups: the experimental group, which received a diaphragmatic stretching technique, or the placebo group, which received a sham-ultrasound procedure. The duration of the technique, the position of participants, and the therapist who applied the technique were the same for both treatments. Participant assessment (cervical range of movement, lumbar flexibility, flexibility of the posterior chain, and rib cage and abdominal excursion) was performed at baseline and immediately after the intervention by a blinded assessor.

**Results:**

The mean between-group difference [95% CI] for the ribcage excursion after technique at xiphoid level was 2.48 [0.97 to 3.99], which shows significant differences in this outcome. The remaining between-group analysis showed significant differences in cervical extension, right and left flexion, flexibility of the posterior chain, and ribcage excursion at xiphoid level (p<0.05) in favor of the experimental group.

**Conclusion:**

Diaphragm stretching generates a significant improvement in cervical extension, right and left cervical flexion, flexibility of the posterior chain, and ribcage excursion at xiphoid level compared to a placebo technique in healthy adults.

TClinical Trials Identifier:NCT01753726

## BULLET POINTS

- Diaphragmatic stretching improved cervical movement and lumbar flexibility.
- Diaphragmatic stretching increased flexibility of the posterior chain.
- After diaphragmatic stretching, ribcage movement increased at xiphoid level.

## Introduction

The dynamic mobility of an articulated chain is determined by the range of the individual joint movements and the muscular properties, defining the range of motion capacity^1^. Muscular chains are composed of gravitational muscles that work synergistically in the maintenance of the standing position. It has been described that the shortening of a muscle creates compensation in the adjacent and also in distant muscles^2^.

The diaphragm is recognized as the primary muscle of respiration that plays an important role in breathing and physiological regulation. It is formed by a central trefoil-shaped tendon that blends superiorly with the fibrous pericardium^3^. The abdominal and thoracic cavities on which the diaphragm action takes place are also involved in postural stability and control. Several studies^4^ ^,^ ^5^ have found a relationship between the activity of the human diaphragm and intercostal muscles and both respiratory and postural functions.

From a biomechanical point of view, the equilibrium of the spine is achieved by a local and a global system of muscle engagement. The stabilizing muscles with insertion or origin at vertebrae (multifidus, transversus abdominis, diaphragm, internal oblique) provide intersegmental stability, whereas the longer trunk muscles (erector spinae, rectus abdominis) are dedicated to general movement^6^. Hence, the local system, where the diaphragm plays an important role, performs an action of stabilization and posture.

Over the last few decades, numerous studies^7^ ^,^ ^8^ have been conducted on the effects of stretching and provided evidence of increased muscle control, flexibility, and range of motion. Although such studies have traditionally focused on muscles of the lower extremities and yielded high-quality research, the biomechanical and structural characteristics of the diaphragm imply an additional difficulty. Techniques aimed at the diaphragm have been used to increase movement in the rib cage and the spine^9^ ^,^ ^10^.

Some evidence supports^11^ a relationship between trunk muscle activity and posterior chain muscle movement. Different studies^12^ ^-^ ^14^ have used stretching techniques including diaphragm stretching for spinal pain relief, improving the posture^12^, stability^13^, and the length of the posterior muscle chain^14^. However, few studies have explored the effect of stretching techniques on diaphragm and spine kinematics. Taking into account the complex structure of the diaphragm and its important role in the postural chain^2^, we were prompted to verify the effects of a diaphragm technique on posterior chain muscle kinematics and rib cage and abdominal excursion in healthy subjects.

## Method

### Participants

This study was completed in the laboratory of the Faculty of Health Sciences, University of Granada, Granada, Spain. Asymptomatic volunteers ranging in age from 18 to 60 years were recruited from the general population between June 2012 and January 2015. Participants were excluded if they exhibited history of neck trauma, history of fracture in any part of the body, herniated disk or lumbar protrusion, history of back surgery, significant respiratory or neurological condition, or regular use of analgesic or anti-inflammatory drugs. Those who were pregnant, reported experiencing major psychological stress, or had consumed caffeinated food and/or beverage products within the previous 24 hours were also excluded.

The randomization sequence was drawn up and kept off-site by a statistician who was not aware of the study aims, using a random number generator in blocks of eight with no stratification. The randomization schedule was delivered, in a sealed envelope, to a research assistant who assigned participants to the groups and organized appointments for the participants by phone. Each subject signed an informed consent statement prior to involvement in the study. Approval for the study was obtained from Ethics Committee of the University of Granada, Granada, Spain (ID number DF0037UG) and the procedures conducted in accordance with the Declaration of Helsinki of 1975. The name of the public trials registry is www.clinicaltrials.gov and the registration number NCT01753726.

### Outcome measures

The study assessor who collected the outcome measurements was blinded to the study hypotheses and group allocation.

### Anthropometric measures

All subjects completed the same tests before and after the intervention. For descriptive purposes, anthropometric measurements were taken at baseline. Body mass was measured in kilograms (Kg) to the nearest 0.1 Kg on a calibrated digital medical scale (Seca 843, Switzerland). Height was measured in centimeters (cm) to the nearest 0.5 cm via a standard wall-mounted stadiometer.

### Chain muscle kinematics

#### Cervical range of motion

A Baseline Bubble Inclinometer (Fabrication Enterprises Inc., White Plains, NY, USA) was used to measure the active range of motion of the cervical spine. The measurements were performed in two planes of movement, lateral flexion (frontal plane) on the right and left side and flexion-extension (sagittal plane). The subject was seated comfortably on a chair. The inclinometer was placed on the top of his/her head, and the subject was asked to move his/her head as far as possible in each movement. A comparison of radiographs and inclinometer measures showed excellent correlations (r<0.9997, P<0.05)^15^. The standard values of cervical extension in healthy subjects of 30-39 years are 36-102 degrees, for left lateral flexion 20-60 degrees and for right lateral flexion 27-62 degrees^16^.

#### Schober’s test

Schober’s test is a trunk flexion test to evaluate lumbar flexibility. While the subject was in the standing position, marks were made on the midpoint between the posterior superior iliac spines and 10 cm above this point. The 10 cm distance was then compared to the distance between the same two marks when the subject was in the forward flexed position. Elongation of 5 cm or more between the two marks during forward flexion is considered to be normal lumbar spine movement^17^. The validity of Schober’s test against radiographs was found to be strong (r=0.90) to moderate (r=0.68). The intraclass (r=0.96) and interclass (r=0.90) reliability was found to be excellent^18^.

#### Finger-to-floor test

In the finger-to-floor test (FFT), subjects stood on a stool and flexed the trunk forward to reach as far as possible with both hands, without bending their knees^19^. The distance (cm) between the level of the stool and the middle finger was measured by the therapist. FFT has high reliability and sensitivity scores^19^.

#### Abdominal and rib cage excursion measures

Abdominal and ribcage measurements can be used as an evaluative method for diaphragmatic breathing excursion to quantify possible alterations in thoracic capacity and abdominal and chest wall compliance as achieved by all expiratory and inspiratory muscles^20^. By recording the abdomen and ribcage excursion with a measuring tape over the second intercostal space (axillary level), xiphoid process, and midpoint between the xiphoid process and umbilicus (abdominal level), competency in diaphragmatic breathing can be demonstrated by a reduction in ribcage excursion^20^. These indirect measurements have an intra-rater reliability of 0.96-0.98 and an inter-rater reliability of 0.84-0.87 with correlation coefficients not less than 0.84^20^ ^,^ ^21^.

### Experimental procedure

Subjects were randomly allocated by selection of sealed envelope into one of two groups – an experimental group or a placebo group. After all the measures were taken, subjects were led to another room where they received the diaphragmatic technique or the placebo intervention. Subjects were then taken back to the first room for the post-treatment measures.

**Figure 1:**
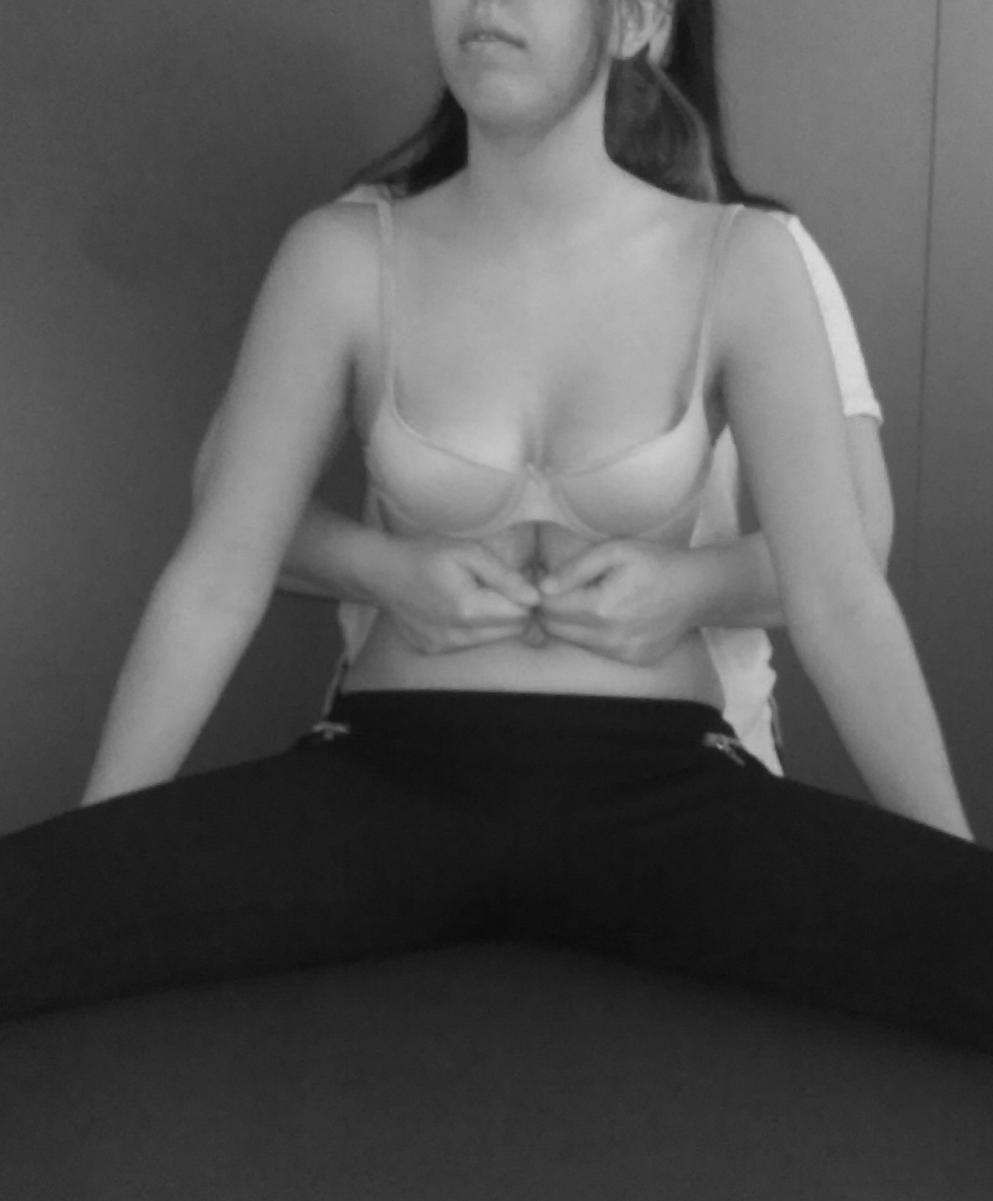
Diaphragm stretching technique.

The stretching of the diaphragm technique was executed as described previously by Chaitow et al.^22^. Each subject was positioned seated erect. The therapist stood behind the subject and passed his hands around the thoracic cage, carefully introducing fingers under the costal margins. The subject rounded the trunk slightly in order to relax the rectus abdominis (Figure 1). When the subject exhaled, the therapist grasped the lower ribs and costal margin and eased the hands caudally. The stretching was performed once and the tension was maintained for 5-7 minutes.

In the placebo group, disconnected ultrasound was applied in the same position for 7 minutes as placebo treatment. The patients had to be seated erect, and the ultrasound was applied in the costal margins.

### Statistical analysis

Data were initially analyzed with regard to their statistical distribution using the Shapiro-Wilks W test. The demographic data and initial assessment results were compared using the t-test with SPSS software, version 17.0 (Statistical Package for the Social Sciences, SPSS Inc., Chicago, IL, USA). The sample size in the current study was powered to detect statistical differences between the 2 groups with 85% power based on a previous pilot study. The t-test for paired samples was used to compare the results of the assessment before and after treatment for parametric data. The Wilcoxon signed rank test was used to perform the above-mentioned comparisons for non-parametric data. The independent t-test and the Mann–Whitney U-test were used to conduct analyses between groups for parametric and non-parametric data, respectively. The alpha level was set at 0.05.

## Results

**Figure 2:**
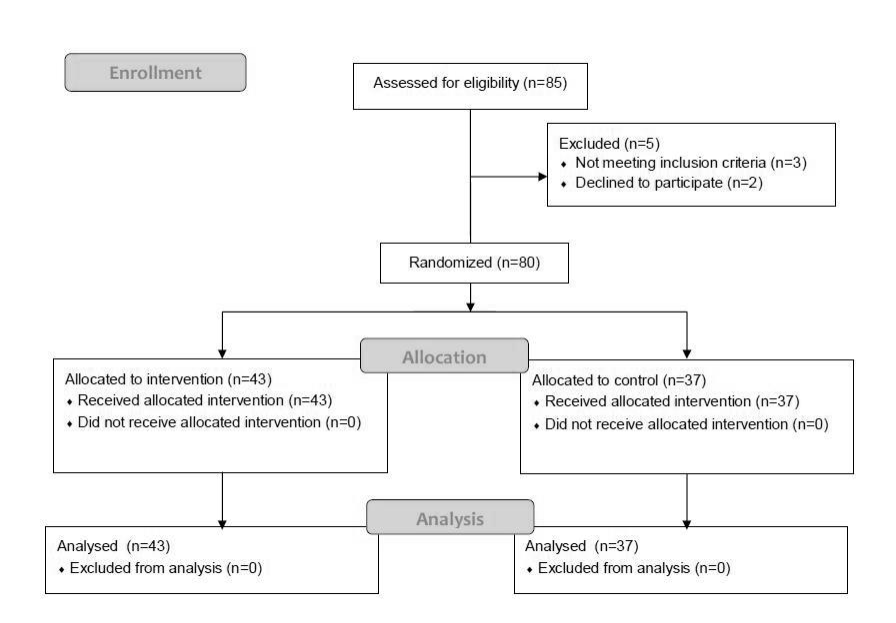
Flow diagram of the progress through the phases of the randomized trial.

The flow of participants through the trial is shown in Figure 2.

Baseline characteristics (Table 1) of both groups were similar although the stretching group had comparatively fewer men, 19 (44.18%) vs. 15 (40.54%). They also had very similar body mass index (BMI) values (23.26±3.3 vs. 23.02±3.36).

**Table 1:** Baseline characteristics of stretching and control group participants.

	Stretching group (n=43)	Control group (n=37)
Sex n (% males)	19 (44.18)	15 (40.54)
Age (years) Mean±SD	36.33±15.93	37.4±15.82
Height (cm) Mean±SD	167±0.83	169±0.99
Weight (kg) Mean±SD	65.22±12.59	66.5±12.10
BMI (kg/cm^2^) Mean±SD	23.26±3.31	23.02±3.36
Smokers n (%)	22 (51.16)	19 (51.35)

Baseline characteristics between groups in the primary outcomes are provided in Table 2, with no significant differences between groups in any of the primary variables (p>0.05).

**Table 2:** Primary outcomes at baseline.

	Stretching group (n=43)	Control group (n=37)
Cervical range of movement		
Flexion (degrees)	46.21±9.36	49.07±6.66
Extension (degrees)	53.14±11.02	55.19±8.02
Right lateral flexion (degrees)	40.35±7.59	41.67±7.07
Left lateral flexion (degrees)	40.51±6.19	43.52±7.05
Schober’s test (cm)	14.52±1.06	14.28±1.08
Finger-to-floor test (cm)	4.66±6.76	3.37±5.24
Rib cage excursion		
Axillary level (cm)	3.89±2.50	3.83±1.59
Xiphoid level (cm)	4.30±2.41	4.69±2.08
Abdominal level (cm)	0.10±2.87	–0.74±1.68

Data are expressed as mean±SD.

In the diaphragm stretching group, significant changes were found between pre- and post-intervention measurement variables in between-group analysis (Table 3).

**Table 3:** Primary outcomes at baseline and post-technique.

	Stretching group (n=43)	P-value	Control group (n=37)	P-value	Mean between-group difference (95% CI)	Between-groups p value
Cervical range of movement						
Flexion Pre-technique Post-technique	46.21±9.31 51.51±7.62	p<0.001^**^	49.07±6.61 50.00±6.72	0.379	1.51 [–2.06 to 5.09]	0.402
Extension Pre-technique Post-technique	53.14±11.0 59.3±9.9	p<0.001^**^	55.19±8.0 55.00±6.35	0.852	4.3 [0.006 to 8.59]	0.050^*^
Right lateral flexion Pre-technique Post-technique	40.35±7.5 44.42±6.51	p<0.001^**^	41.67±7.01 41.30±5.90	0.646	3.12 [0.01 to 6.23]	0.049^*^
Left lateral flexion Pre-technique Post-technique	40.51±6.11 46.98±6.2	p<0.001^**^	43.52±7.01 43.7±5.20	0.832	3.27 [0.37 to 6.17]	0.028^*^
Schober’s test Pre-technique Post-technique	14.52±1.05 15.01±1.03	p<0.001^**^	14.27±1.07 14.33±1.10	0.376	0.67 [0.15 to 1.19]	0.011^*^
Finger-to-floor test Pre-technique Post-technique	4.66±6.76 3.37±5.80	0.001^**^	3.37±5.21 3.33±5.32	0.646	0.039 [–2.72 to 2.8]	0.978
Rib cage excursion						
Axillary level Pre-technique Post-technique	3.89±2.50 4.27±1.87	0.352	3.83±1.59 3.87±1.43	0.895	0.34 [–0.43 to 1.23]	0.347
Xiphoid level Pre-technique Post-technique	4.30±2.40 6.93±3.45	p<0.001^**^	4.69±2.08 4.44±2.36	0.582	2.48 [0.97 to 3.99]	0.002^*^
Abdominal level Pre-technique Post-technique	0.10±2.87 –0.12±2.57	0.885	–0.74±1.68 0.741±1.66	0.020^*^	-0.75 [-1.86 to 0.35]	0.181

Data are expressed as the mean±SD.

* Significant differences p<0.05.

** Significant differences p≤0.001.

For the control group, significant differences were found at abdominal level (p=0.02).

The between-group analysis showed significant differences in cervical extension, right and left flexion, flexibility of the posterior chain, and ribcage excursion at xiphoid level (p<0.01).

## Discussion

The main purpose of the study was to determine whether the application of a diaphragm stretching resulted in changes in posterior chain muscle kinematics and ribcage and abdominal excursion in healthy subjects. The results supported the hypothesis that a manual technique improves the variables measured by Schober’s test and the finger-to-floor test, as well as cervical mobility and xiphoid level ribcage excursion immediately after the technique. No significant differences were observed in rib cage excursion at axillary and abdominal level between groups. It is normal that the highest changes were observed at the xiphoid level, the nearest to the diaphragm, where the stretching was performed. Due to the anatomical access to the diaphragm, an anterior approach was performed. The biomechanical relationship between the diaphragm and other structures supports the hypothesis that a diaphragm technique can have a repercussion on other distant structures, as previously reported^2^ ^,^ ^14^. Therefore, we have included the variables related to mobility of the posterior chain in this study.

The control group showed significant changes in abdominal excursion, which can be explained by the relaxing posture adopted.

The sample of subjects included in the groups was representative of a generally homogeneous adult population (similar percentage of smokers, age range, and BMI values). This homogeneity reduced the probability of obtaining confounding factors that might affect the value of our results.

Muscular chain contractions can cause changes in the range of motion in other distant structures/muscles, because muscles work synergistically in the same chain^2^. It has been suggested that the shortening of a muscle creates compensation in adjacent and also in distant muscles^2^. From an anatomical viewpoint, the diaphragm is a muscle with a central trefoil-shaped tendon that blends superiorly with the fibrous pericardium. The origins of the diaphragm are placed in the crura from the bodies of the lumbar vertebrae, the arcuate ligaments, the costal margins, and the xiphoid^23^. Therefore, the biomechanical relationship between the diaphragm and other structures support the hypothesis that diaphragm stretching can have a repercussion on other distant structures^2^, improving the flexibility of the posterior chain muscle and spine structure mobility.

Our findings are consistent with those previously reported by other authors^22^ ^,^ ^24^ who have explored the use of different techniques of manual therapy as an option to increase the mobility of the spine in healthy subjects. Saíz-Llamosas et al.^24^ suggested that using a cervical myofascial induction technique increases cervical flexion, extension, and left lateral-flexion.

Among the various types of manual therapy, stretching techniques have been used in several studies on the effects of stretching and evidenced increased muscle control, flexibility, and range of motion^8^ ^,^ ^25^. Additionally, stretching techniques have been suggested to be efficient in promoting respiratory variables such as maximal respiratory pressures, thoracic expansion, and abdominal mobility^26^. An interesting finding of our study is that diaphragm stretching improves cervical motion. Similarly, Kasunich^27^ found that an abnormal functioning of supporting distal structures can induce biomechanical disturbances in proximal areas.

The analysis of pre-to-post stretching values provided important data on posterior chain muscle kinematic changes after diaphragm stretching. From a therapeutic approach, diaphragm stretching can be used as an effective therapeutic tool with an immediate response. The results obtained are important in a therapeutic context because it is evidenced that obtaining and maintaining range of motion is very important and a key factor in injury prevention.

Some limitations need to be mentioned, such as the absence of follow-up in order to determine how long the changes in kinematics were maintained and the application in healthy subjects. Due to the anatomical access to the diaphragm, an anterior approach was performed and only the costal portion of the diaphragm was lengthened, but our results have shown that there is a positive effect in the main outcome measures. The short length of the therapeutic session (5-7 minutes) could be one of the limitations of this study. However, previous studies^2^ ^,^ ^14^ have investigated the immediate effects of manual techniques with beneficial results.

Diaphragm stretching is a safe and well-tolerated technique with an immediate significant effect. Further studies are needed to evaluate the applicability of this technique in symptomatic populations. This research could be used in other case scenarios and future research, not only to prevent injury. Diaphragm stretching could also be added to traditional interventions in the treatment of whiplash, which can affect cervical, thoracic, and lumbar regions as well and the rib cage.

## Conclusions

Diaphragm stretching generated a significant improvement in posterior chain muscle kinematics measured by Schober’s test, the finger-to-floor test, cervical range of motion, and ribcage excursion at xiphoid level immediately after the technique. In contrast, the placebo technique showed no pre- or post-technique differences in any of the measures. The between-group analysis showed significant differences in cervical right and left flexion, flexibility of the posterior chain, and ribcage excursion at xiphoid level.
